# Imposing control on self-assembly: rational design and synthesis of a mixed-metal, mixed-ligand coordination cage containing four types of component†
†Electronic supplementary information (ESI) available: Synthesis of the cage complex; bond distances and angles around the metal ions; COSY and DOSY NMR data; and high resolution electrospray mass spectral expansion for the cage. CCDC 1425635. For ESI and crystallographic data in CIF or other electronic format see DOI: 10.1039/c5sc03526k



**DOI:** 10.1039/c5sc03526k

**Published:** 2015-10-14

**Authors:** Alexander J. Metherell, Michael D. Ward

**Affiliations:** a Department of Chemistry , University of Sheffield , Sheffield S3 7HF , UK . Email: m.d.ward@sheffield.ac.uk

## Abstract

Retrosynthetic analysis of a [M_16_L_24_]^32+^ coordination cage shows how it can be assembled rationally, in a stepwise manner, using a combination of kinetically inert and kinetically labile components. Combination of the components of *fac*-[Ru(L^ph^)_3_](PF_6_)_2_, Cd(BF_4_)_2_ and L^naph^ in the necessary 4 : 12 : 12 stoichiometry afforded crystals of [Ru_4_Cd_12_(L^ph^)_12_(L^naph^)_12_]X_32_ (X = a mono-anion) in which the location of the two types of metal ion [Ru(ii) or Cd(ii)] at specific vertices in the metal-ion array, and the two types of bridging ligand (L^ph^ and L^naph^) along specific edges, is completely controlled by the synthetic strategy. The incorporation of four different types of component at pre-determined positions in a coordination cage superstructure represents a substantial advance in imposing control on the self-assembly of complex metallosupramolecular entities.

## Abstract 2

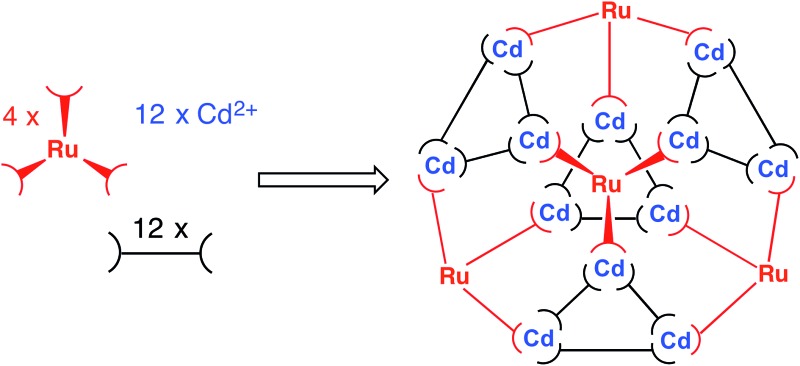
A stepwise assembly method, using a combination of kinetically inert and kinetically labile components, allows formation of a coordination cage based on four types of component with each component directed to a specific site.

## Introduction

The syntheses of different types of metal/ligand polyhedral coordination cage provide examples of purely serendipitous reactions, in which the structure of the product was entirely unexpected, to rationally-designed reactions in which geometric rigidity of ligands and pronounced stereochemical preferences of metal ions can be exploited.^1^ Most syntheses of cage complexes lie somewhere in between these extremes. Typically an initial cage synthesis using self-assembly involves some serendipity, especially if flexible components are involved:^1*c*^ but then sensible variations and extensions of this initial result, by making for example minor alterations to the ligand structure, allow predictable modifications to be made. Thus Fujita and co-workers have shown how changing the bend angle in a rigid bis(pyridyl) ligand changes the radius of curvature (and hence the nuclearity) of a Pd(ii)-based ‘nanosphere’;^2^ Nitschke and co-workers have made simple length extensions to rigid ditopic ligands to increase the size of the resulting M_4_L_6_ tetrahedra.^3^

**Fig. 1:**
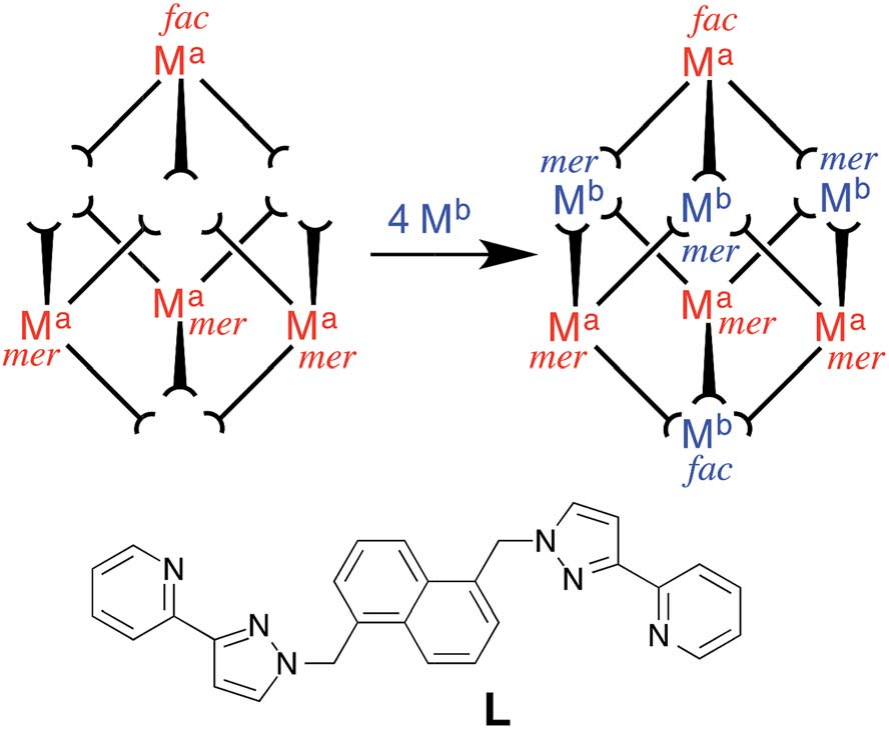
Example of stepwise preparation of heterometallic cages from a mixture of kinetically inert units [(M^a^)L_3_]^2+^ and additional labile ions (M^b^)^2+^ in a 4 : 4 stoichiometry (from ref. 4).

We are seeking to bring control and predictability to the preparation of large coordination cages by selecting mononuclear metal/ligand fragments of coordination cages and pre-preparing these in isolation using kinetically inert metal ions such as Ru(ii) and Os(ii).^4^ These mononuclear ‘fragments’ are based on ditopic ligands and therefore have pendant binding sites at which cage assembly can propagate by coordination of additional labile ions which connect the fragments into a complete heterometallic cage. An illustration of our initial examples is in Fig. 1: four equivalents of a kinetically stable, mononuclear [(M^a^)L_3_]^2+^ complex (M^a^ = Ru, Os, with the required *fac* : *mer* ratio of geometric isomers needed to complete the final product) are combined with labile metal ions (M^b^)^2+^ (M^b^ = Co, Cd) which assemble with the pendant binding sites to form the heterometallic cages [(M^a^)_4_(M^b^)_4_L_12_]^16+^ in which metal ion types alternate around the cage periphery. This rational, stepwise synthesis – based on the initial serendipitous formation of the octanuclear cage – allowed specific types of functional behaviour associated with the metal ions such as redox activity (Ru, Os) and long-lived luminescence (Os) to be incorporated into the cage superstructures.^4^ More generally, it also allows the number of different types of component involved in an assembly to increase from two (one type of metal ion + one type of ligand) to three, with the two types of metal ion introduced separately.

We report here how we have extended this principle a significant step further, with preparation of a hexadecanuclear cage^5,6^ containing not just two different types of metal ion at different vertices of the metal polyhedron, but also containing a mixture of two different types of ligand along different edges. The ability to incorporate four different types of component at specific, pre-determined sites in the assembly provides an unusual and significant level of synthetic control.^7^ The location of all four types of component in the cage superstructure follows from the synthetic procedure, which in turn is derived from dissection of the cage into its component parts in a kind of ‘retrosynthetic analysis’. Given the increasing importance of exploiting the host/guest chemistry of cages for their functional behaviour^7^ – from drug delivery^8^ to catalysis^9^ – the ability to exert control over the self-assembly process, and introduce different types of functionality at specific sites in the superstructure, is likely be of considerable value.

## Results and discussion

**Fig. 2:**
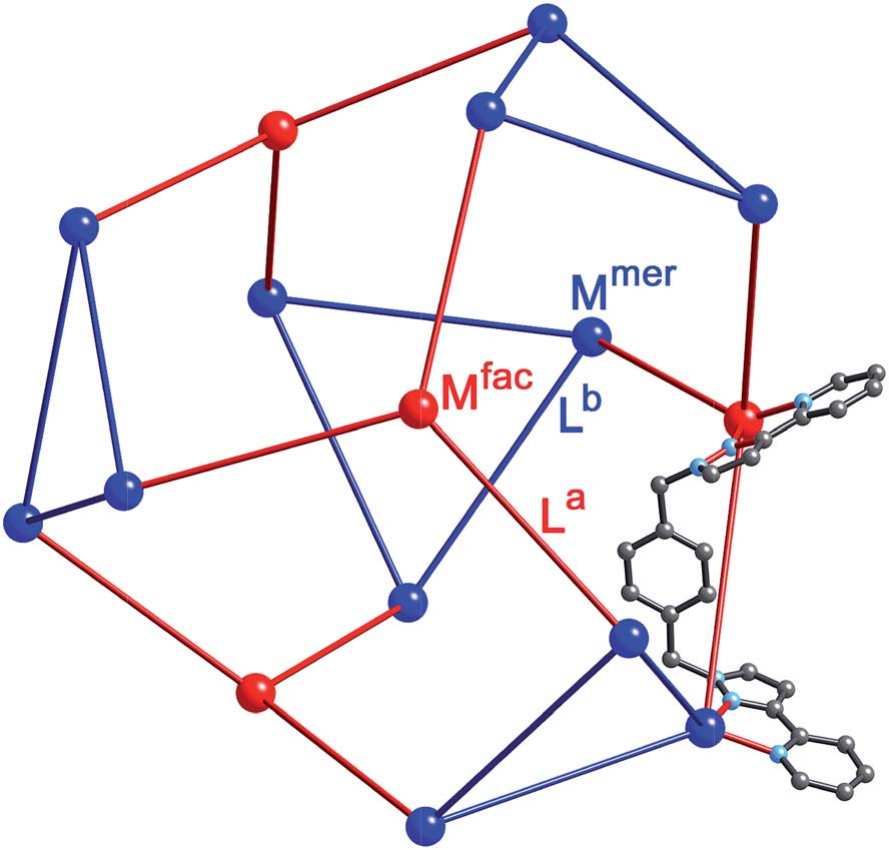
Representation of the core structure of [Cd_16_(L^ph^)_24_](ClO_4_)_32_ (ref. 5) with one bridging ligand included. All metal sites are Cd(ii) but the two different types of geometric isomer are colour-coded: *fac* tris-chelate metals are in red (M^*fac*^, see main text) and *mer* tris-chelate metals are in blue (M^*mer*^). Likewise the two ligand environments are L^a^ in red, and L^b^ in blue (see main text).

**Fig. 3:**
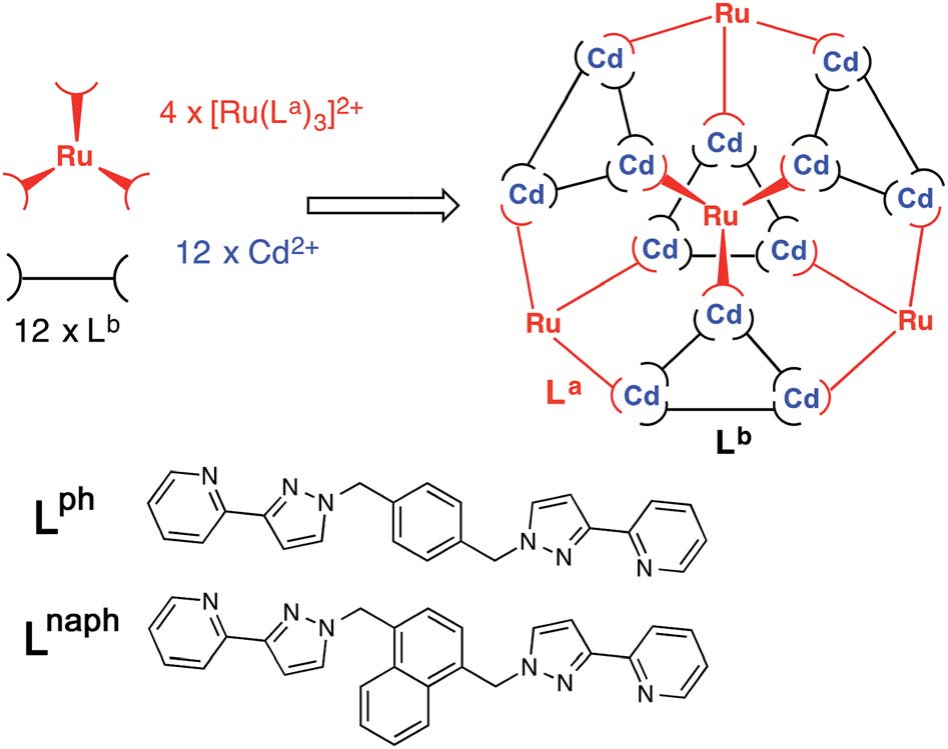
Sketch of the stepwise synthetic strategy used in this work to prepare the heterometallic, mixed-ligand cage: *viz.* combination of pre-formed *fac*-[Ru(L^a^)_3_]^2+^ (red), additional labile Cd^2+^ ions (blue), and free ligand (L^b^, black) in a 4 : 12 : 12 ratio to give hexadecanuclear [Ru_4_Cd_12_(L^a^)_12_(L^b^)]^32+^ with L^a^ = L^ph^ and L^b^ = L^naph^.

The target cage type for stepwise assembly is the [M_16_L_24_]^32+^ cage (Fig. 2) in which the metal ions lie at the vertices of a twisted tetra-capped truncated tetrahedral array, with a bridging ligand spanning every edge.^5,6^ The first examples of these, [M_16_(L^ph^)_24_]^32+^ (M = Zn, Cd), were the unexpected products arising from combination of the metal salts with L^ph^ in the required 2 : 3 ratio; as with many of our larger cages, the cage seemed to be stabilised by extensive aromatic π-stacking between ligand fragments around the cage periphery.^5*a*^ However these cages proved to be unstable in solution, with crystals of redissolved [Cd_16_(L^ph^)_24_](ClO_4_)_32_ rearranging over a period of weeks in solution to the smaller trigonal prismatic cage [Cd_6_(L^ph^)_9_](ClO_4_)_12_.^5*b*^ Replacing L^ph^ with L^naph^ (Fig. 3) resulted in isostructural cages in which the increased surface area available for π-stacking from the naphthyl groups rendered the cages stable in solution with no rearrangement being detectable.^6^

We chose this cage type as the target for our synthesis as (i) it is the most complex structure in our cage family,^1*c*^ and (ii) is known to form with either of two types of ligand and any of several types of metal ion,^5,6^ increasing the possibilities for controlling the complexity of the product in a predictable way. A ‘retrosynthetic analysis’ of the cage allows us to identify how best to dissect the structure into component parts for a stepwise synthesis. Firstly, it is obvious that there are two types of metal ion environment. The four pseudo-octahedral metal ions (M^*fac*^) that constitute the ‘caps’ over the hexagonal faces of the truncated tetrahedron all have a *fac* tris-chelate geometry provided by the three chelating pyrazolyl–pyridine units. The remaining twelve metal ions (M^*mer*^) all have a *mer* tris-chelate coordination geometry, and describe four triangular M_3_L_3_ cyclic helical fragments: connection of four of these affords the truncated tetrahedral core of the cage. Thus the 16 metal ions therefore split into a set of four (M^*fac*^, isolated from one another; red in Fig. 2) and a set of 12 (M^*mer*^, connected to one another in sets of three; blue in Fig. 2). Secondly, the ligands likewise can be split into two types: those that connect a *fac* to a *mer* vertex (L^a^), and those connecting two *mer* vertices in the cyclic helicate triangular panels (L^b^). There are 12 of each type of ligand. This subdivision of metal ions and ligands into different types is shown in Fig. 2.^5*b*^

So: which type of metal vertex should be pre-prepared as a kinetically inert subcomponent for a rational, stepwise assembly? Our initial choice of metal ions is Ru(ii) for the kinetically inert vertices, given the straightforward and well-established synthesis and purification of stable tris-chelate complexes as their pure *fac* and *mer* isomers;^10,11^ and Cd(ii) for the kinetically labile vertices to facilitate ^1^H NMR analysis. If we prepare *mer*-[RuL_3_]^2+^ units (blue in Fig. 2) and try build the complete cage around these there are two immediate problems. Firstly, it is not possible for two Ru(ii) units to be connected to one another by a single bridging ligand: each *mer*-[RuL_3_]^2+^ unit will necessarily connect to three Cd(ii) ions. Thus each triangular (M^*mer*^)_3_L_3_ unit could only contain one Ru(ii) ion with the other two necessarily being Cd(ii), and it is likely that these would be positionally disordered around the triangle. Inclusion of one Ru(ii) ion into each of the four (M^*mer*^)_3_L_3_ units of a completed cage at a random position will generate multiple isomers of the metal skeleton which rather defeats the point of the ‘predictable’ synthesis that we are trying to achieve. Secondly, the *mer*-[RuL_3_]^2+^ unit would include ligands which end up in two different types of position in the cage (L^a^ and L^b^). Thus, if we prepare a complex such as *mer*-[Ru(L^ph^)_3_]^2+^ as a building block, we have lost the possibility of introducing a chemically different ligand at each of sites L^a^ and L^b^.

The alternative possibility is far more logical and attractive, *viz.* to use Ru(ii) at the M^*fac*^ sites and Cd(ii) at the M^*mer*^ sites. This requires preparation of homoleptic *fac*-[Ru(L^a^)_3_]^2+^ units in which L^a^ could be either L^ph^ or L^naph^. Each of these will necessarily bind to three Cd(ii) ions. The remaining bridging ligands L^b^, which connect the Cd(ii) ions around the triangular cyclic helicate units, can then be added separately to the reaction. There is no problem with them being chemically different from L^a^, as long as both L^a^ and L^b^ are interchangeable and support the same cage type (as L^ph^ and L^naph^ do).^5,6^

Our analysis of the structure therefore suggests that a cage containing two different metal ions at predictable positions, *and* two different ligand types at predictable positions, can be assembled from four pre-prepared *fac*-[Ru(L^ph^)_3_]^2+^ units, twelve additional Cd(ii) ions, and twelve additional ligands L^naph^ to give the heteronuclear, heteroleptic cage [Ru_4_Cd_12_(L^ph^)_12_(L^naph^)_12_]^32+^ (Fig. 3). In this assembly M^*fac*^ = Ru; M^*mer*^ = Cd; L^a^ = L^ph^; L^b^ = L^naph^ and there is no possibility for disorder of metal ions or ligand types between sites as long as the [Ru(L^ph^)_3_]^2+^ units remain stable as the *fac* isomer.

**Fig. 4:**
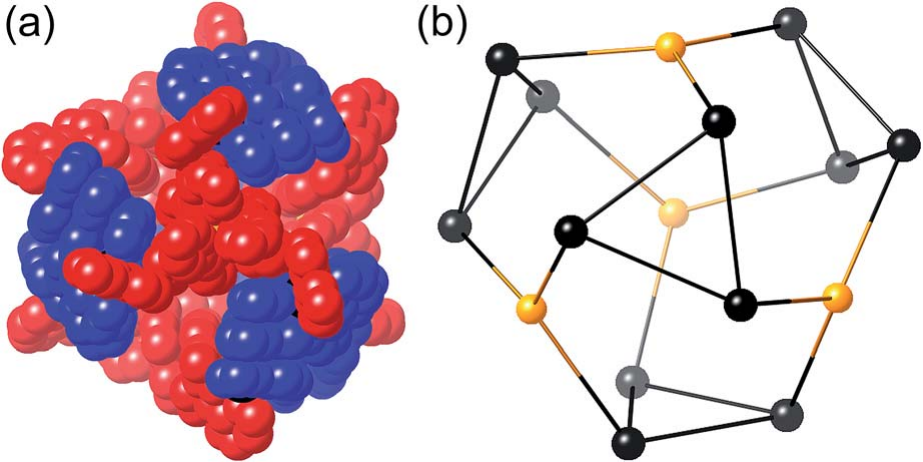
Two views of the crystal structure of [Ru_4_Cd_12_(L^ph^)_12_(L^naph^)_12_](PF_6_)_7_(BF_4_)_25_. (a) The entire complex cation in spacefilling view (L^naph^ shown in blue, L^ph^ shown in red); (b) arrangement of metal ions in the Ru_4_Cd_12_ core (Ru – yellow, Cd – black).

**Fig. 5:**
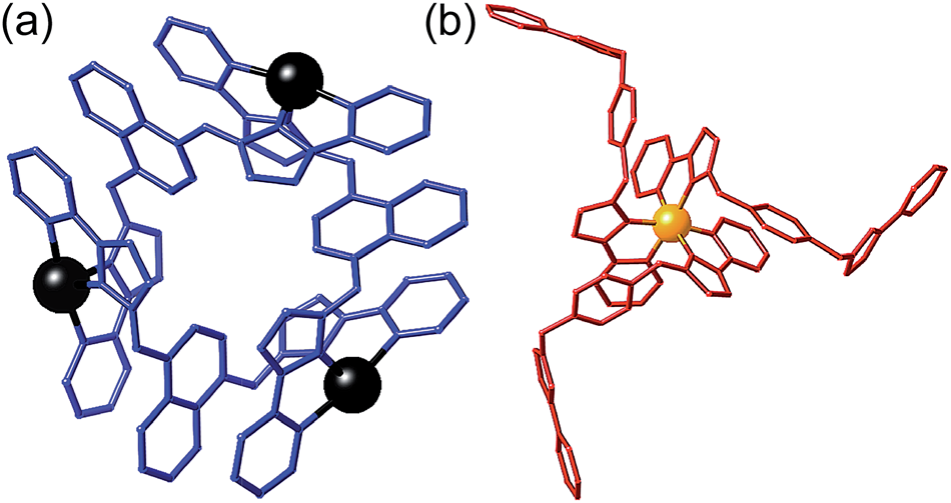
Fragments from the crystal structure of [Ru_4_Cd_12_(L^ph^)_12_(L^naph^)_12_] (PF_6_)_7_(BF_4_)_25_ (same colouring scheme as Fig. 4). (a) A [Cd_3_(L^naph^)_3_]^6+^ triangular cyclic helicate unit; (b) a *fac*-[Ru(L^ph^)_3_]^2+^ unit.

**Fig. 6:**
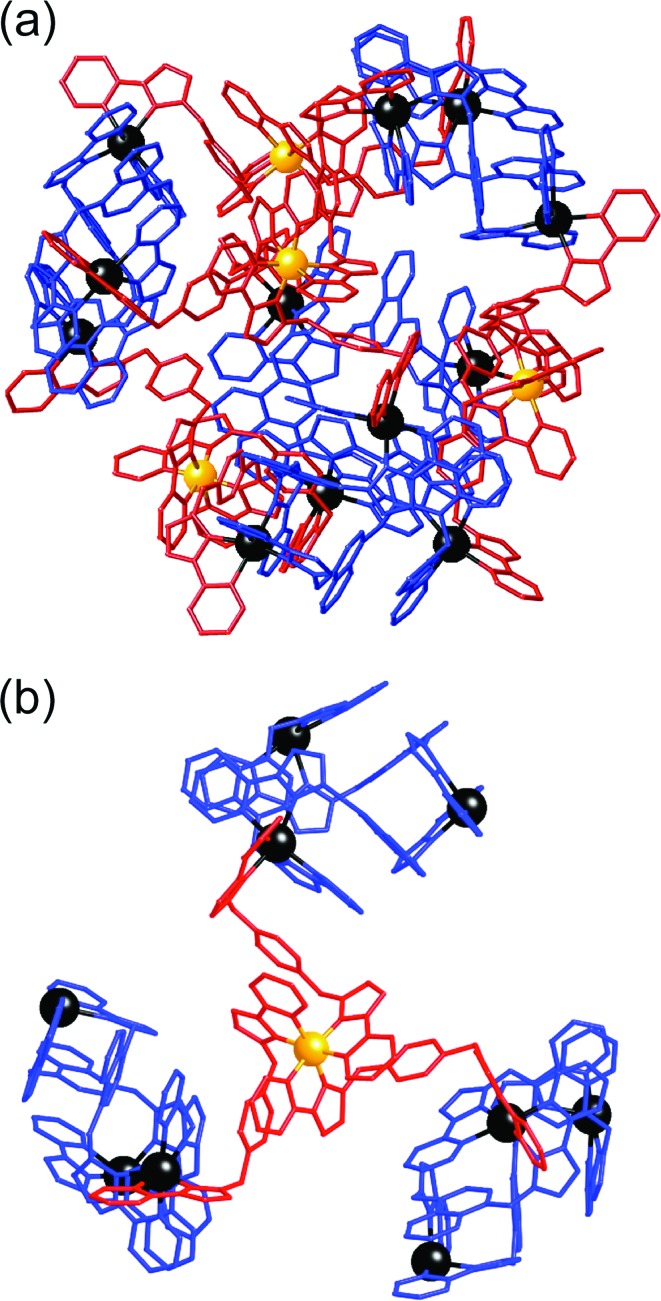
Left: View of the complete complex cation of [Ru_4_Cd_12_(L^ph^)_12_(L^naph^)_12_](PF_6_)_7_(BF_4_)_25_, (same colouring scheme as Fig. 4). Right: Partial view of the complex, emphasising how each *fac*-[Ru(L^ph^)_3_]^2+^ vertex is connected to three [Cd_3_(L^naph^)_3_]^6+^ units.

**Fig. 7:**
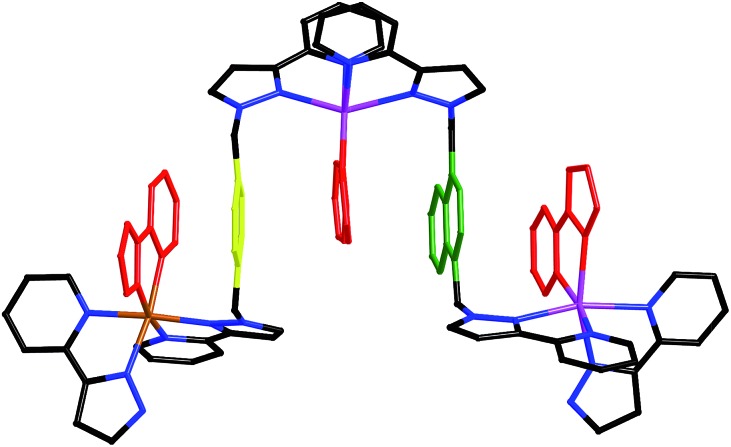
Five-layer aromatic stack in the structure of [Ru_4_Cd_12_(L^ph^)_12_(L^naph^)_12_](PF_6_)_7_(BF_4_)_25_ with electron-rich phenyl and naphthyl rings in yellow and green, respectively, and electron deficient pyrazolyl–pyridine units in red. Cd – purple; Ru – orange.

The necessary kinetically inert Ru(ii) complex *fac*-[Ru(L^ph^)_3_](PF_6_)_2_ was available from previous work.^12^ The required combination of *fac*-[Ru(L^ph^)_3_](PF_6_)_2_, Cd(BF_4_)_2_ and L^naph^ in a 1 : 3 : 3 ratio in nitromethane was prepared and required gentle heating for 1 h to enable all components to dissolve (see ESI† for details). After filtration of the mixture through a membrane filter, recrystallisation over several weeks by slow diffusion of diisopropyl ether vapour into the nitromethane solution with afforded the product as X-ray quality yellow crystals. The crystals were extremely sensitive to solvent loss, with crystallinity deteriorating rapidly once removed from the mother liquor. After several attempts a suitable crystal was mounted and a crystal structure determined as the desired cage complex [Ru_4_Cd_12_(L^ph^)_12_(L^naph^)_12_](PF_6_)_7_(BF_4_)_25_ (Fig. 4–7). We found that the presence of a mixture of anions made it easier to obtain X-ray quality single crystals.

The complex crystallised in the space group *P*1, with one entire cage occupying the asymmetric unit and therefore being crystallographically unique (over 800 independent non-hydrogen atoms).‡
‡Crystallographic data for [Ru_4_Cd_12_(L^ph^)_12_(L^naph^)_12_](PF_6_)_7_(BF_4_)_25_·MeNO_2_·H_2_O. C_625_H_509_B_25_Cd_12_F_142_N_145_O_3_P_7_Ru_4_, *M* = 15 036.86 g mol^–1^, triclinic, space group *P*1, *a* = 30.518(2), *b* = 31.449(2), *c* = 54.722(4) Å; *α* = 81.113(4), *β* = 76.270(4), *γ* = 68.114(4)°; *U* = 47 215(6) Å^3^, *Z* = 2, *ρ*_calc_ = 1.058 g cm^–3^, *T* = 100(2) K, *μ*(Cu-Kα) = 3.378 mm^–1^. 286 355 reflections with 2*θ*_max_ = 100° were merged to give 93 244 independent reflections (*R*_int_ = 0.15). Final *R*1 [for data with *I* > 2*σ*(*I*)] = 0.164; w*R*2 (all data) = 0.436. Data were collected on a Bruker D8 Venture diffractometer at the University of Sheffield. After integration of the raw data, and before merging, an empirical absorption correction was applied (SADABS)^14^ based on comparison of multiple symmetry-equivalent measurements. The structure was solved by direct methods and refined by full-matrix least squares on weighted *F* ^2^ values for all reflections using the SHELX suite of programs.^15^ The crystal exhibited the usual problems of this type of structure, *viz.* weak scattering due to a combination of poor crystallinity, solvation, and disorder of anions/solvent molecules. The structure and connectivity of the complex cation could nonetheless be unambiguously determined with reasonable precision. Extensive use of geometric restraints on aromatic rings and anions, and restraints on aromatic displacement parameters, were required to keep refinement stable. Solvent molecules that could be modelled satisfactorily were included in the final refinements; large regions of diffuse electron density that could not be modelled (from disordered solvents/counter ions) were removed from the refinement, using the SQUEEZE function in PLATON.^16^ Full details are in the CIF. CCDC deposition number: 1425635. Despite the typical crystallographic problems associated with weak diffraction from large assemblies containing extensive disorder of anions and solvent molecules, which has led to an *R*1 value of 16%, the key features are quite clear. It is immediately obvious that the core structure of the cage is the same as that of the previously reported [M_16_L_24_]^32+^ cages,^5,6^ where sixteen metal ions are arrayed in a tetra-capped truncated tetrahedral array, with M···M separations along the edges lying in the range 9.19–10.31 Å (Fig. 2): of these the Cd···Cd distances lie in the range 9.67–10.31 Å, and the Ru···Cd distances lie between 9.19–9.86 Å. The identities of the Ru(ii) and Cd(ii) ions are obvious from their very different M···N distances: the Ru–N distances are in the range 2.0–2.1 Å whereas the Cd–N distances are in the range 2.3–2.4 (see ESI†), and we can see that the different ions are in their allotted positions with the Ru(ii) ions at all four *fac* sites and the Cd(ii) ions at all twelve *mer* sites. The two different types of ligand are trivial to distinguish as they are chemically different, with the twelve L^ph^ ligands (containing a 1,4-phenylene spacer) all spanning a Ru···Cd edge, and the twelve L^naph^ ligands all spanning the Cd···Cd edges around the Cd_3_ triangles. As is usual with cages based on ligands from this family,^1*c*^ the structure exhibits extensive inter-ligand π-stacking around the periphery involving alternating arrays of electron-rich (naphthyl or phenyl) and electron-deficient (coordinated pyrazolyl–pyridine) groups (Fig. 7). Each five-component stack contains three pyrazolyl–pyridine units as the electron-deficient component, and these alternate with one phenyl and one naphthyl unit as the electron-rich components.

Although many of the anions could not be located from the data due to disorder, those that could be located are close to the cage surface and involved in weak CH···F interactions with the ligands. In particular several anions are located in the windows: both the larger ones in the centres of the Ru_2_Cd_4_ faces, and the smaller ones associated with the Cd_3_ faces (see ESI†). Diffuse electron density inside the cage could not be assigned and is part of what was removed using the SQUEEZE function: so the cage appears empty, but only because its contents were disordered.

**Fig. 8:**
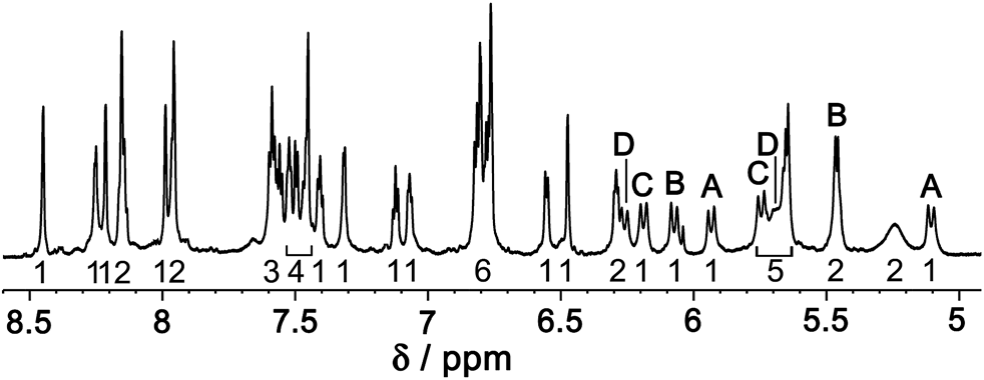
^1^H NMR spectrum (CD_3_NO_2_, 800 MHz) of redissolved crystals of [Ru_4_Cd_12_(L^ph^)_12_(L^naph^)_12_](PF_6_)_7_(BF_4_)_25_. Integers under the signals are integral values (total 42). Labels (A–D) refer to the four pairs of doublets from diastereotopic methylene groups, identified from a COSY spectrum, which confirm the presence of two independent ligand environments with no internal symmetry and equal numbers of each type (see main text).

Several pieces of evidence confirm that the cage is stable in solution and retains its structure. An 800 MHz ^1^H NMR spectrum is consistent with the presence of two independent ligand environments (L^ph^, 20 protons; L^naph^, 22 protons), each with no internal symmetry, corresponding to 42 independent ^1^H signals of equal intensity (Fig. 8). Whilst these could not be assigned individually due to overlap, the number of signals is clearly correct on the basis of integral values, and a COSY spectrum (ESI†) shows the presence of four pairs of doublets from the four inequivalent and diastereotopic CH_2_ groups (two for each ligand type).

A DOSY spectrum (ESI†) clearly confirmed the presence of a single species with a log *D* value (–9.6, with *D* expressed in m^2^ s^–1^) typical of a cage of this size^5b,6^ but quite different from that of any mononuclear species such as [Ru(L^ph^)_3_](PF_6_)_2_ which has a log *D* value of –8.4.^12^ There is no evidence for slow decomposition or rearrangement of the cage in solution, in contrast to the behaviour of homoleptic [Cd_16_(L^ph^)_24_](ClO_4_)_32_.^5*b*^ This may be ascribed partly to the presence of twelve L^naph^ ligands in the ligand set which increase the surface area involved in π-stacking compared to the phenyl rings,^6^ and partly to the presence of the four kinetically inert Ru(ii) centres, which will not undergo dissociation of a chelating ligand under mild conditions – which is the essential first step to rearrangement of a coordinatively saturated complex at room temperature.

**Fig. 9:**
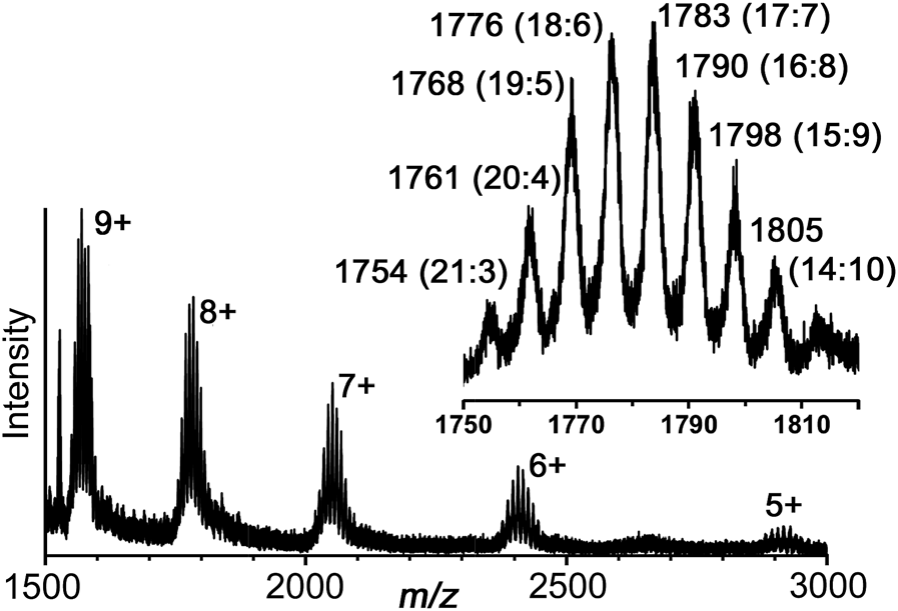
Partial electrospray mass spectrum of redissolved crystals of [Ru_4_Cd_12_(L^ph^)_12_(L^naph^)_12_](PF_6_)_7_(BF_4_)_25_ showing the sequence of signals associated with progressive loss of anions. For each charge, the presence of multiple closely-spaced signals is associated with different combinations of [BF_4_]^–^ and [PF_6_]^–^ anions. The inset shows the expansion of the set of signals around *m*/*z* 1780 for the 8+ ions: the number of [BF_4_]^–^ and [PF_6_]^–^ anions for each is shown in parentheses. Thus, the signal at *m*/*z* 1768 corresponds to {[Ru_4_Cd_12_(L^ph^)_12_(L^naph^)_12_](BF_4_)_19_(PF_6_)_5_}^8+^, *etc.* For high-resolution expansions, see ESI.†

Electrospray mass spectrometry also confirmed the formulation of the cage with a series of peaks corresponding to the species {[Ru_4_Cd_12_(L^ph^)_12_(L^naph^)_12_]_32–*x*_}^*x*+^, *i.e.* the intact hexadecanuclear cation associated with varying numbers of anions (Fig. 9). High-resolution measurements show accurate mass values and isotope patterns that are exactly consistent with the observed structure (see ESI†).

## Conclusions

In conclusion, the stepwise synthetic methodology for preparation of heterometallic cages based on initial preparation of kinetically inert fragments, for which we reported the first examples recently,^11^ has been substantially extended. We have performed a rational two-step synthesis of the hexadecanuclear mixed-metal, mixed-ligand cage [Ru_4_Cd_12_(L^ph^)_12_(L^naph^)_12_](PF_6_)_7_ (BF_4_)_25_, which contains two types of ligand *and* two types of metal ion, all at pre-determined positions within the superstructure that are dictated by the synthesis. This was possible by analysis of the optimal way of separating the structure into (i) regions that can be pre-assembled using a kinetically inert metal ion, and (ii) regions that can be allowed to undergo normal self-assembly using labile components. In particular the use of *fac*-[Ru(L^ph^)_3_](PF_6_)_2_ as the inert component restricts the Ru(ii) ions to the four M^*fac*^ sites, which in turn restricts the associated L^ph^ ligands to the L^a^ sites: and this in turn defines where the Cd(ii) ions and the L^naph^ ligands must go in the final assembly. Given the useful functional behaviour which can be associated with these types of fragment [*e.g.* redox activity from the Ru(ii) ions^4^ and luminescence from the naphthyl groups^13^] this type of controlled synthetic approach will be valuable for synthesis of coordination cages that contain desired functionality at specific sites in the superstructure.
