# Supplementary material for: Imposing control on self-assembly: rational design and synthesis of a mixed-metal, mixed-ligand coordination cage containing four types of component
Source: Chem Sci. 2015 Oct 14;7(2):910–5. doi: 10.1039/c5sc03526k (PMC5530507; doi:10.1039/c5sc03526k)
Supplement: Supplementary file 1 [file SC-007-C5SC03526K-s001.pdf]

Electronic Supplementary Material (ESI) for Chemical Science.
This journal is © The Royal Society of Chemistry 2015

Imposing control on self-assembly: rational design and synthesis of a mixed-metal, mixed-ligand coordination cage containing four types of component

Alexander J. Metherell and Michael D. Ward

Supporting Information

1 Experimental information

2 Bond distances (Å) around the metal ions from the crystal structure

3 High-resolution electrospray mass spectra of selected signals

4 Additional NMR data

5 Additional figures of the crystal structure of $[Ru_4Cd_{12}(L^{ph})_{12}(L^{naph})_{12}](PF_6)_7(BF_4)_{25}$

1. Experimental information

General details

Metal salts and all organic reagents were purchased from Alfa or Sigma-Aldrich and used as received. ^{1}H NMR spectra were recorded on Bruker DRX 500 MHz, Bruker AV-III 400 MHz or AV-1 800 MHz instruments. Low-resolution electrospray mass spectra were recorded on a Micromass LCT instrument. High-resolution electrospray mass spectra were measured on an electron transfer dissociation (ETD) enabled ThermoFisher-Scientific Orbitrap Elite, equipped with an HESI source (ThermoFisher Scientific). UV/Vis absorption spectra were measured on a Cary 50 spectrophotometer.

Preparation of $[Ru_4Cd_{12}(L^{ph})_{12}(L^{naph})_{12}](PF_6)_7(BF_4)_{25}$

To a stirred solution of *fac*-$[Ru(L^{ph})_3](PF_6)_2$ (0.023 g, 0.015 mmol; preparation in ref 1) in nitromethane (10 cm^3) was added $[Cd_3(L^{naph})_3](BF_4)_6$ (0.035 g, 0.015 mmol; preparation in ref. 6, main text). The initially turbid solution became clear after heating at 60 ^{o}C for 1 hour, after which the mixture was passed through a membrane filter. Slow diffusion of di-isopropyl ether into the nitromethane solution yielded the complex as large yellow blocks of X-ray quality, which were separated from the mother liquor and washed with di-isopropyl ether. Yield: 0.022 g, 40 %. ESMS (selected peaks): *m/z* 2835, $([Ru_4Cd_{12}(L^{ph})_{12}(L^{naph})_{12}](BF_4)_{26}(PF_6))^{5+}$; 2348, $([Ru_4Cd_{12}(L^{ph})_{12}(L^{naph})_{12}](BF_4)_{25}(PF_6))^{6+}$; 2000, $([Ru_4Cd_{12}(L^{ph})_{12}(L^{naph})_{12}](BF_4)_{24}(PF_6))^{7+}$. UV/Vis in MeCN [$\lambda_{max}$/nm ($10^{-3}$ ε/M^{-1} cm^{-1})]: 397 (50.3), 287 (577.0), 229 (783.8). Note that the extinction coefficient associated with the Ru-based MLCT absorption at 397 nm is *ca.* four times more intense than for mononuclear *fac*-$[Ru(L^{ph})_3](PF_6)_2$ (ref. 12, main text).

2. Bond distances (Å) around the metal ions from the crystal structure

Cd(1)-N(11K)	2.298(9)	Cd(2)-N(11D)	2.275(8)
Cd(1)-N(11E)	2.313(8)	Cd(2)-N(31C)	2.310(8)
Cd(1)-N(22E)	2.327(8)	Cd(2)-N(42C)	2.316(10)
Cd(1)-N(22K)	2.342(10)	Cd(2)-N(42V)	2.329(10)
Cd(1)-N(22B)	2.345(8)	Cd(2)-N(22D)	2.380(8)
Cd(1)-N(11B)	2.358(8)	Cd(2)-N(31V)	2.418(11)
Cd(4)-N(11Q)	2.287(9)	Cd(6)-N(42E)	2.278(10)
Cd(4)-N(41N)	2.298(9)	Cd(6)-N(31F)	2.283(9)
Cd(4)-N(31D)	2.317(11)	Cd(6)-N(31E)	2.295(11)
Cd(4)-N(22Q)	2.347(9)	Cd(6)-N(42F)	2.338(9)
Cd(4)-N(42D)	2.360(11)	Cd(6)-N(11H)	2.357(11)
Cd(4)-N(31N)	2.377(10)	Cd(6)-N(22H)	2.381(12)
Cd(9)-N(11L)	2.248(10)	Cd(10)-N(42W)	2.231(11)
Cd(9)-N(11M)	2.291(11)	Cd(10)-N(31M)	2.298(10)
Cd(9)-N(22L)	2.302(9)	Cd(10)-N(22T)	2.315(11)
Cd(9)-N(42A)	2.309(10)	Cd(10)-N(11T)	2.321(9)
Cd(9)-N(22M)	2.356(10)	Cd(10)-N(31W)	2.351(13)
Cd(9)-N(31A)	2.374(11)	Cd(10)-N(42M)	2.373(11)
Cd(11)-N(42G)	2.258(13)	Cd(12)-N(42P)	2.246(10)
Cd(11)-N(11R)	2.284(10)	Cd(12)-N(42R)	2.269(13)
Cd(11)-N(42O)	2.315(12)	Cd(12)-N(31R)	2.306(12)
Cd(11)-N(31O)	2.329(10)	Cd(12)-N(31P)	2.316(12)
Cd(11)-N(22R)	2.340(9)	Cd(12)-N(31X)	2.341(13)
Cd(11)-N(31G)	2.412(12)	Cd(12)-N(42X)	2.374(12)
Cd(14)-N(31J)	2.257(13)	Cd(15)-N(42I)	2.221(16)
Cd(14)-N(22C)	2.287(10)	Cd(15)-N(42T)	2.283(13)
Cd(14)-N(31Q)	2.297(11)	Cd(15)-N(31L)	2.283(9)
Cd(14)-N(42Q)	2.303(13)	Cd(15)-N(42L)	2.329(9)
Cd(14)-N(11C)	2.321(10)	Cd(15)-N(31T)	2.364(10)
Cd(14)-N(42J)	2.399(12)	Cd(15)-N(31I)	2.388(12)
Cd(16)-N(11F)	2.245(14)	Cd(7)-N(11O)	2.293(9)
Cd(16)-N(22F)	2.297(14)	Cd(7)-N(11P)	2.319(10)
Cd(16)-N(31K)	2.312(12)	Cd(7)-N(22P)	2.322(10)
Cd(16)-N(42K)	2.330(11)	Cd(7)-N(42U)	2.339(10)
Cd(16)-N(42S)	2.337(16)	Cd(7)-N(22O)	2.359(9)
Cd(16)-N(31S)	2.41(2)	Cd(7)-N(31U)	2.360(12)
Ru(3)-N(11V)	2.022(9)	Ru(5)-N(22J)	2.036(9)
Ru(3)-N(22V)	2.032(8)	Ru(5)-N(11G)	2.043(9)
Ru(3)-N(22A)	2.038(9)	Ru(5)-N(31H)	2.046(11)
Ru(3)-N(42B)	2.075(8)	Ru(5)-N(22G)	2.058(10)
Ru(3)-N(31B)	2.083(9)	Ru(5)-N(11J)	2.074(9)
Ru(3)-N(11A)	2.089(9)	Ru(5)-N(42H)	2.083(9)
Ru(8)-N(22S)	2.029(10)	Ru(13)-N(11I)	1.988(13)
Ru(8)-N(22U)	2.059(9)	Ru(13)-N(22N)	2.053(10)
Ru(8)-N(11W)	2.072(10)	Ru(13)-N(11N)	2.071(12)
Ru(8)-N(22W)	2.083(9)	Ru(13)-N(22X)	2.074(9)
Ru(8)-N(11U)	2.100(10)	Ru(13)-N(11X)	2.098(11)
Ru(8)-N(11S)	2.108(8)	Ru(13)-N(22I)	2.109(11)

3. High-resolution electrospray mass spectra of selected signals

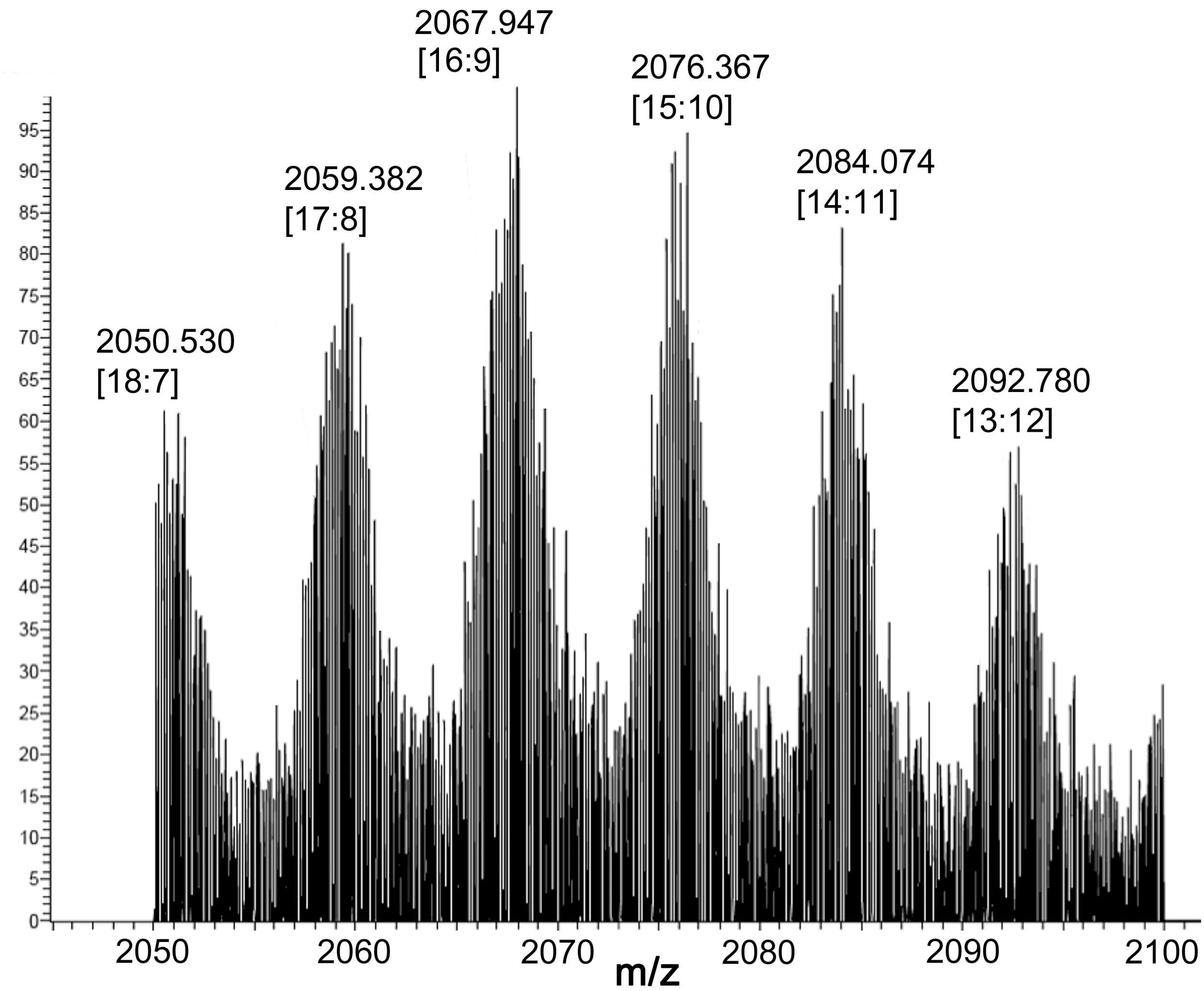

Figure S1 High resolution ES mass spectrum of the cage in the region showing 7+ ions, showing the series of signals for $\{[Ru_4Cd_{12}(L^{ph})_{12}(L^{naph})_{12}](BF_4)_x(PF_6)_y\}^{7+}$ where $x + y = 25$. For each cluster the values in square brackets [x:y] are the numbers of each type of anion.

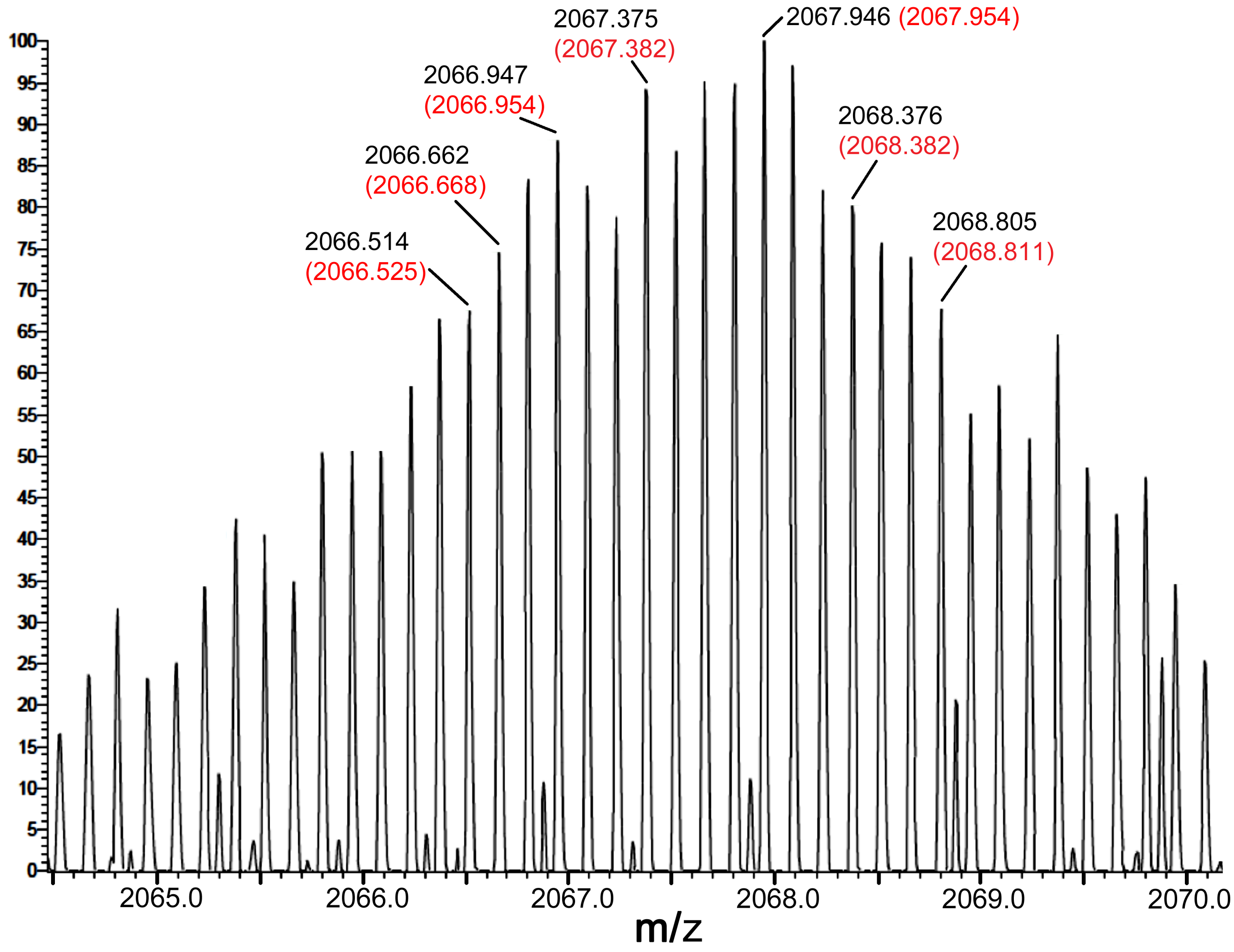

Figure S2 Expansion of the region in Fig. S1 corresponding to the species $\{[Ru_4Cd_{12}(L^{ph})_{12}(L^{naph})_{12}](BF_4)_{16}(PF_6)_9\}^{7+}$, showing the 1/7 mass unit isotopic spacing and the good agreement with calculated *m/z* values (shown in red).

4. Additional NMR data

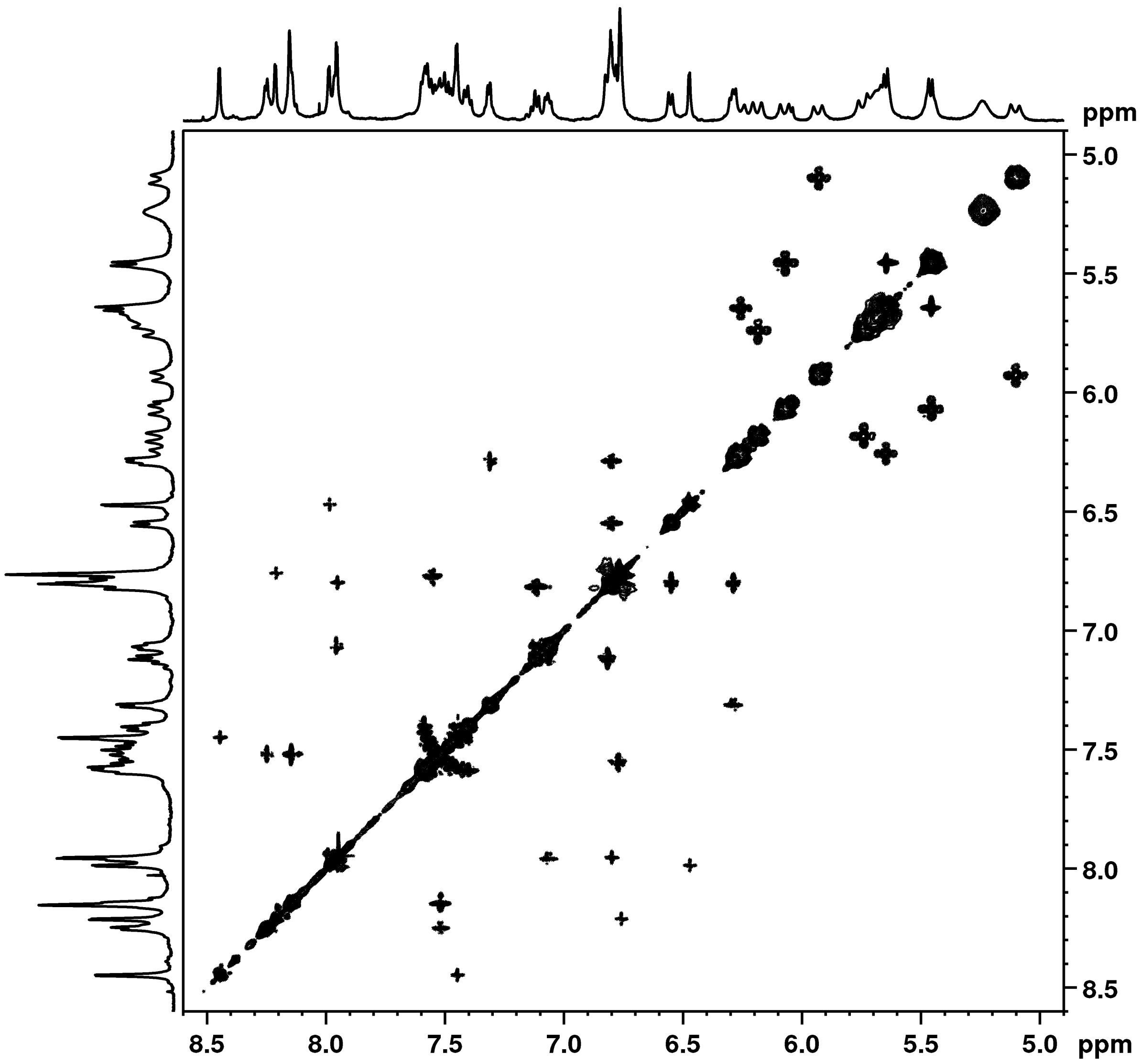

Figure S3 COSY spectrum (500 MHz, 298K) of $[Ru_4Cd_{12}(L^{ph})_{12}(L^{naph})_{12}](PF_6)_7(BF_4)_{25}$ in CD_3NO_2.

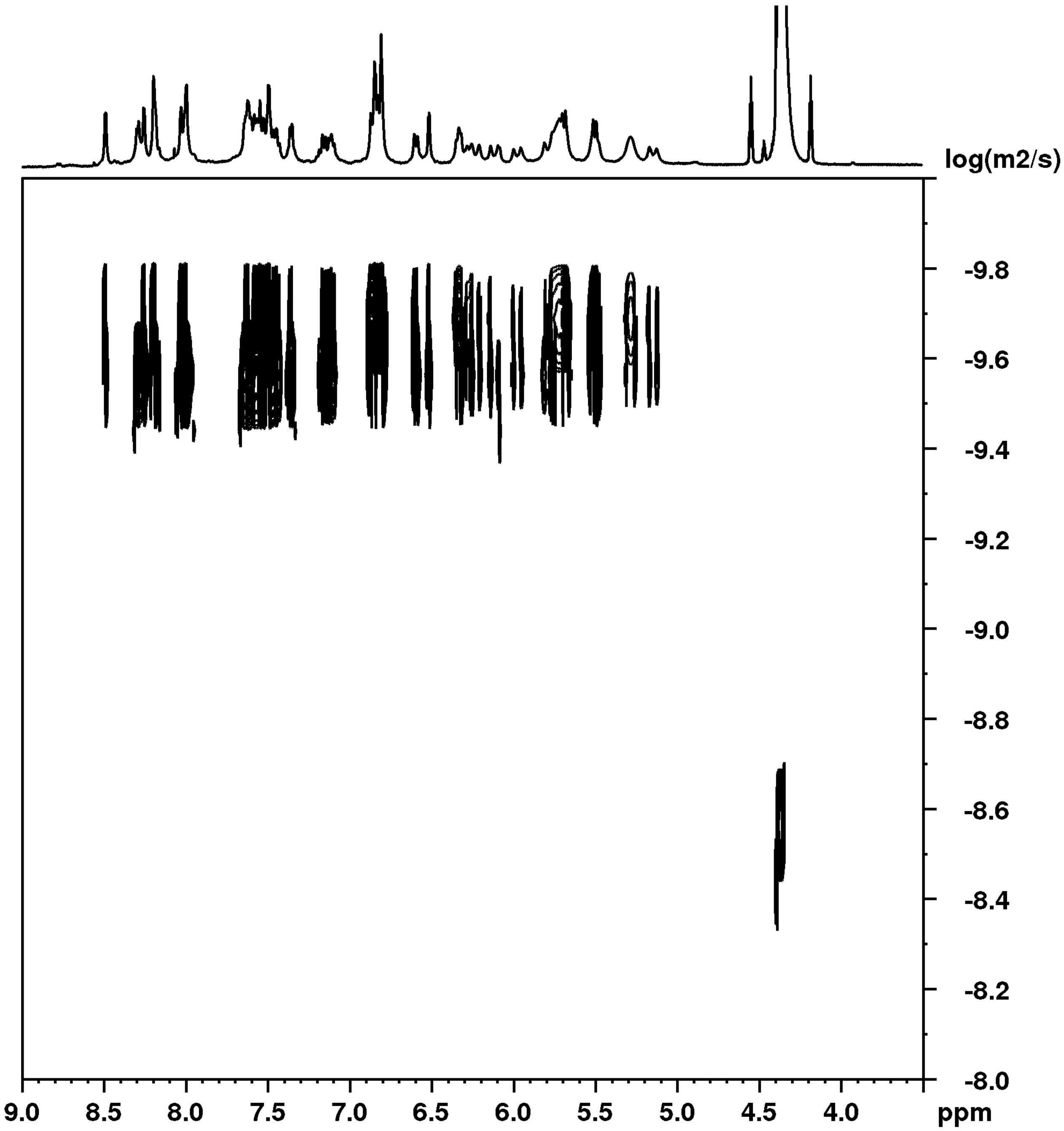

Figure S4 DOSY spectrum (400 MHz, 298K) of $[Ru_4Cd_{12}(L^{ph})_{12}(L^{naph})_{12}](PF_6)_7(BF_4)_{25}$ in CD_3NO_2.

5. Additional figures of the crystal structure of $[Ru_4Cd_{12}(L^{ph})_{12}(L^{naph})_{12}](PF_6)_7(BF_4)_{25}$

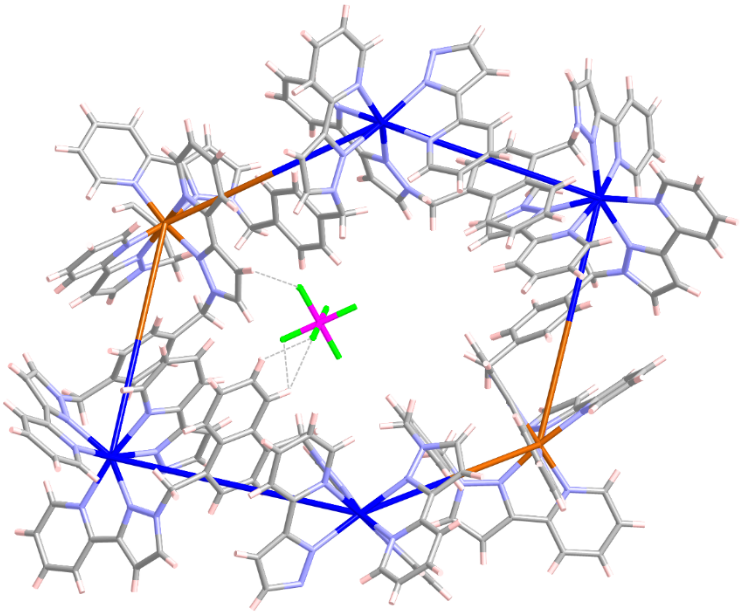

Figure S5 View of one of the large Ru_2Cd_4 windows of $[Ru_4Cd_{12}(L^{ph})_{12}(L^{naph})_{12}](PF_6)_7(BF_4)_{25}$, with an associated $[PF_6]^-$ anion forming C-H…F contacts with the surface of the cage.

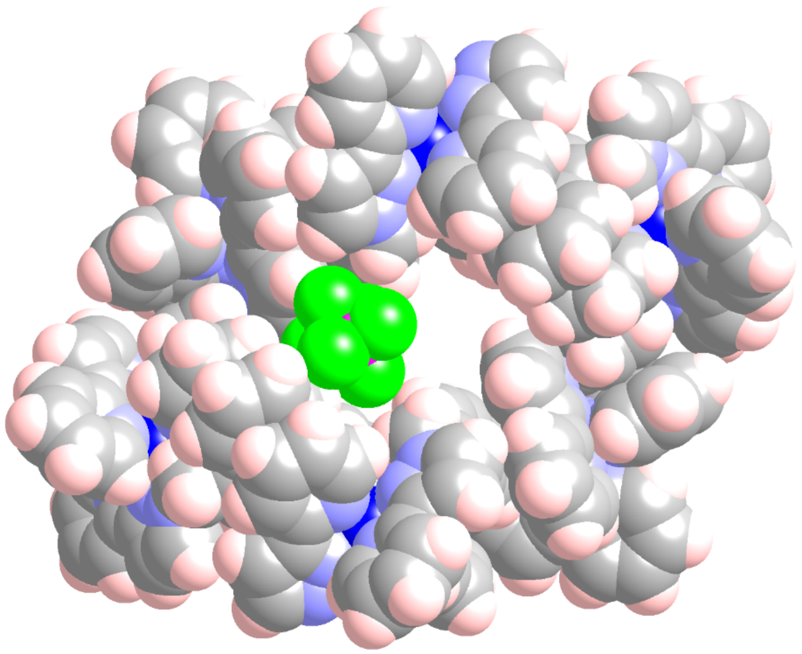

Figure S6 Space-filling version of Fig. S5

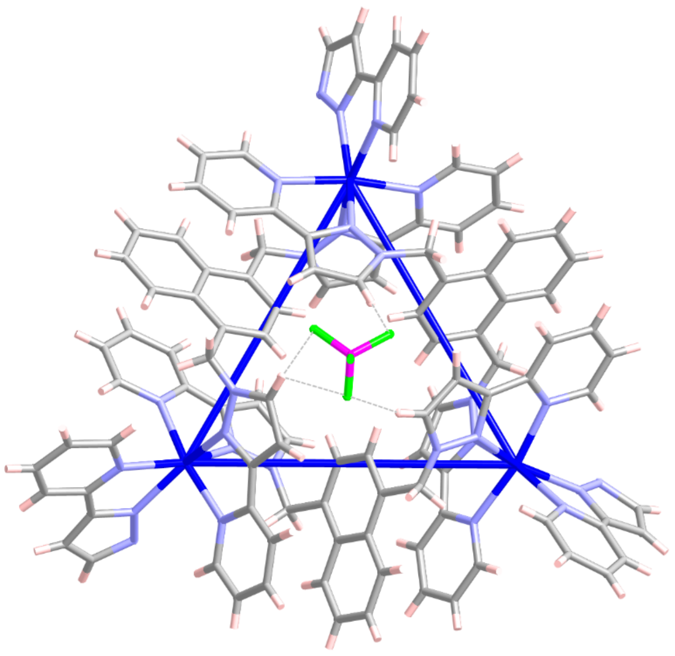

Figure S7 View of one of the Cd_3 windows, with an associated $[BF_4]^-$ anion forming C-H…F contacts with the surface of the cage.

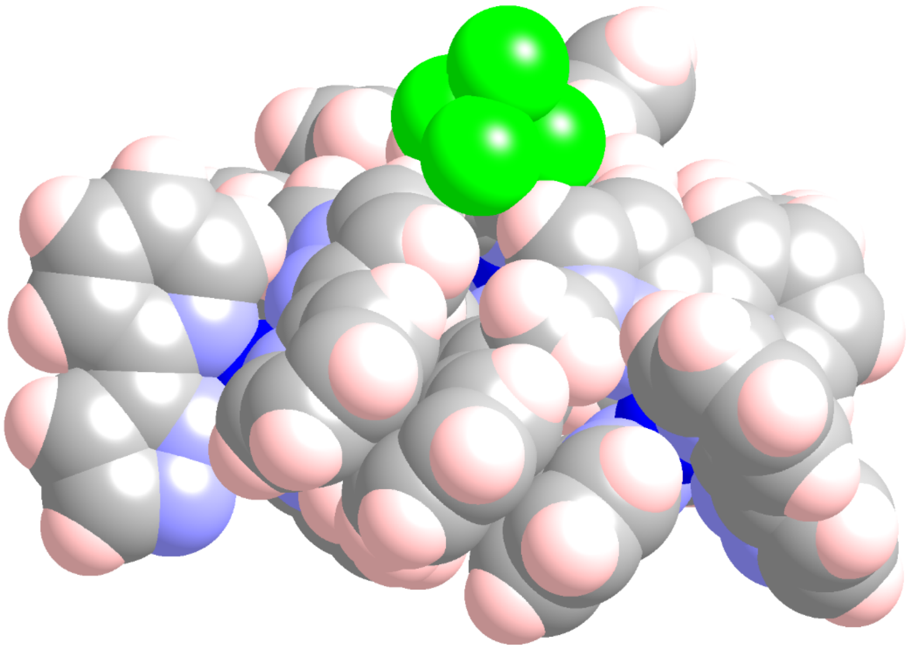

Figure S8 Spacefilling view of Fig. S7 from a different orientation.
